# Directional Selection Causes Decanalization in a Group I Ribozyme

**DOI:** 10.1371/journal.pone.0045351

**Published:** 2012-09-18

**Authors:** Eric J. Hayden, Christian Weikert, Andreas Wagner

**Affiliations:** 1 Department of Bioengineering, Stanford University, Stanford, California, United States of America; 2 Institute of Evolutionary Biology and Environmental Studies, University of Zurich, Zurich, Switzerland; 3 The Swiss Institute of Bioinformatics, Lausanne, Switzerland; 4 The Santa Fe Institute, Santa Fe, New Mexico, United States of America; CRG, Spain

## Abstract

A canalized genotype is robust to environmental or genetic perturbations. Canalization is expected to result from stabilizing selection on a well-adapted phenotype. Decanalization, the loss of robustness, might follow periods of directional selection toward a new optimum. The evolutionary forces causing decanalization are still unknown, in part because it is difficult to determine the fitness effects of mutations in populations of organisms with complex genotypes and phenotypes. Here, we report direct experimental measurements of robustness in a system with a simple genotype and phenotype, the catalytic activity of an RNA enzyme. We find that the robustness of a population of RNA enzymes decreases during a period of directional selection in the laboratory. The decrease in robustness is primarily caused by the selective sweep of a genotype that is decanalized relative to the wild-type, both in terms of mutational robustness and environmental robustness (thermodynamic stability). Our results experimentally demonstrate that directional selection can cause decanalization on short time scales, and demonstrate co-evolution of mutational and environmental robustness.

## Introduction

Biological robustness is the persistence of phenotypes to perturbations [1]. Phenotypes can be robust against external perturbations (environmental robustness) or genetic perturbations (mutational robustness). The ubiquity of robustness in natural systems is extensively documented, and has lead to the hypothesis that robustness itself is subject to evolutionary change [2]–[6]. A canalized genetic system is one that has evolved an optimal and robust phenotype, such that it is buffered against environmental perturbations and/or genetic mutations. Canalization is the expected consequence of long periods of stabilizing selection on well-adapted phenotypes. Its opposite is *decanalization*, which may occur during periods of directional selection, when genotypes with new phenotypes sweep through a population. A decanalized genotype is less robust, and phenotypically more variable than its canalized ancestor. Decanalization has important implications, both for the evolution of novel forms in biology [7]–[9], and as a cause of human disease [10], [11] because most novel phenotypes are deleterious [12]–[16].

The evolution of canalization requires an increase in the robustness of a population over time. To determine a change in canalization experimentally requires the measurement of robustness in evolutionarily related populations. Specifically, for mutational robustness, a direct experimental approach would determine the effects of random mutations for an ancestral and a derived population. Unfortunately, ancestral populations are rarely accessible in the wild. Moreover, in living organisms, the effects of mutation are usually intertwined with those of selection, because only surviving organisms can be studied in the first place. This makes analysis of mutational effects difficult [17]. Severe bottlenecking can be used in mutation accumulation studies to promote the power of drift, and to decrease the power of selection. However, these experiments are labor intensive, especially if mutation accumulation is required on two different genetic backgrounds (ancestral and derived). This has restricted the study of canalization through the application of mutation accumulation experiments [18], [19]. Another approach is to engineer a specific mutation with major effects in order to intentionally decanalize populations that are evolutionarily related. The phenotypic variability that is exposed by the mutation is taken as a measure of canalization because it is caused by the previously hidden genetic variation that accompanies mutational robustness. These types of experiments have been done, for example, for inbred lines of *Drosophila* and in evolutionarily related strains of *E. coli* [20], [21]. They are also labor intensive, and thus limit the number of mutations that can be studied. A more fundamental complication of this approach is that hidden variation can accumulate over time, even if canalization has not changed [7]. Attempts have also been made to validate the apparent canalization of natural genotypes, such as sequences encoding RNA secondary structures, by comparison to ancestral sequences or computational null models [22]–[25]. The results have been contradictory, highlighting the need for direct experimental approaches.

Here, we study robustness in a phenotype where random mutations are easily introduced, and where deleterious effects of mutations can be directly measured. Our study system is a self-splicing intron embedded in the tRNA^Ile^ of the bacterium *Azoarcus* [26]. This RNA enzyme or ribozyme can be removed from its tRNA context, and it can be used to catalyze the cleavage of an exogenous RNA oligonucleotide substrate *in vitro*. This biochemical activity is the phenotype we study. Random nucleotide substitutions can be introduced into this ribozyme through a mutagenic PCR protocol [27]. The activity of a ribozyme population before and after the introduction of mutations can be used to directly measure mutational robustness.

In a previous publication, we subjected populations of this ribozyme to a period of directional selection by chemically modifying its RNA substrate, and selecting for the most active ribozyme variants on this new substrate [28], [29]. During this period of directional selection, the mean activity of the population increased. We identified a high fitness genotype within the population, referred to as Azo*, which has four nucleotide substitutions relative to the wild-type ribozyme. During directional selection, the frequency of the Azo* variant increased more rapidly than that of any other genotype, and comprised about 25% of the population at the end of our experiment (eight rounds of mutagenesis and selection). The Azo* genotype had significantly increased biochemical activity relative to the wild-type, and accounted for most of phenotypic change of the population. In the present study, we determined how this period of directional selection affected mutational robustness of the evolving population as a whole, and how the selective sweep of the Azo* genotype affected this robustness.

## Results and Discussion

### Directional Selection Results in Decreased Mutational Robustness

To determine the effect of directional selection on mutational robustness, we determined the mutational robustness of populations of RNA enzymes taken from before and after directional selection. Specifically, we constructed many “mutational neighbors” for each of these two populations by subjecting the parental population to a mutagenic PCR protocol [27]. This protocol produces one mutation per individual per PCR, on average. Therefore, the most common type of variant was one mutation away from a genotype in the parental population. We expected that these random mutations would cause a decrease in activity of the neighbors relative to the parental populations. To estimate the mutational robustness *R* of the ribozyme’s catalytic phenotype, we determined the ratio of the activity of the mutational neighbors to the activity of their parental population. A higher ratio means a higher mutational robustness. We note that in order to ensure equal mutation rates between “mutational neighbors” from each population, we synthesized them side-by-side and even used the same PCR “master mix” to ensure that all reagents were in identical concentrations during the mutagenic PCR.

**Figure 1:**
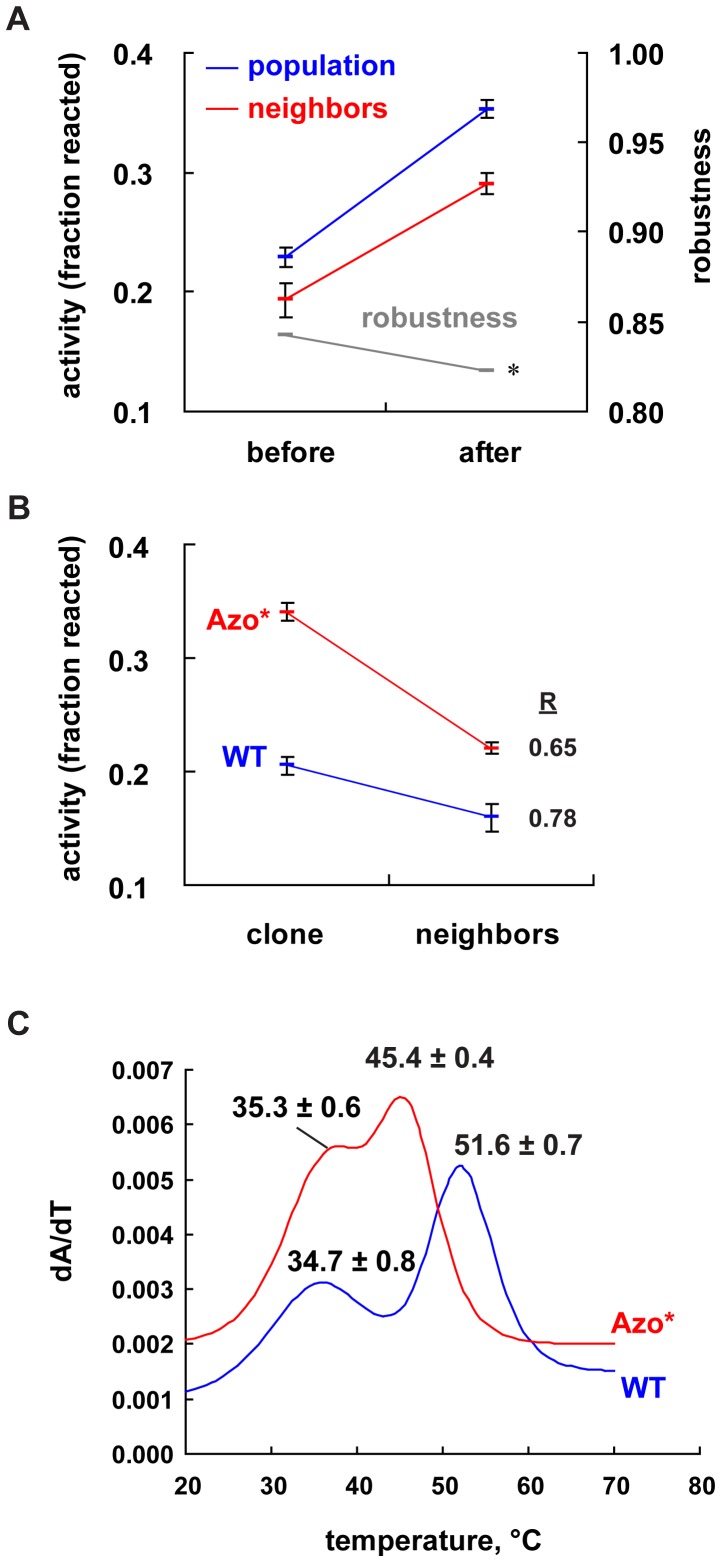
Evidence of decanalization. Activities are measured as the fraction of the ribozyme sample that reacts under our experimental conditions (1h at 37°C, 25 mM MgCl_2_, 30 mM EPPS pH 7.5). Error bars represent 95% confidence intervals. (A) The activities are shown for ribozyme samples taken before and after directional selection. Samples were prepared with high-fidelity PCR (“population”, blue) or with a mutagenic PCR protocol (“neighbors”, red). Robustness is calculated as the ratio of the neighbors’ activity to the population activity at each time (grey). The asterisk indicates that the decrease in robustness is significant (P = 0.016). (B) Mutational robustness of the Azo* (red) and wild-type (“WT”, blue) genotypes. Robustness *R* is measured as the ratio of the activities of the neighbors to the clones for each genotype. (C) Evidence of decreased environmental robustness (thermodynamic stability) of the Azo* genotype. UV absorbance was measured in 10 mM sodium cacodylate buffer (pH 7.5). The plot shows an example of the best-fit curve to data plotted as a derivative of the UV-absorbance (dA/dT) as a function of temperature (°C) for the Azo* (red) and wild-type (blue) ribozymes. The values above each peak indicate the mean and 95% confidence intervals from at least seven replicates (eight for Azo*). Curves were produced using the program Global Melt Fit (http://www.jhu.edu/~chem/draper).

The results show that directional selection leads to decanalization in the form of decreased mutational robustness (Fig. 1A). The parental population increased its activity (positive “slope” of the blue line), which demonstrates that it adapted to the directional selection pressure. The red line connects the mean activities of the two mutant populations, both of which lie below those of their parental population. This demonstrates that mutagenesis indeed lowers the average activity in both populations. What is more, the random mutations have a greater deleterious effect in the population after directional selection, which is visually apparent in the slope of the red line being smaller than that of the blue line (significant interaction in two-way ANOVA, P = 0.016, n = 12). This means that the population before directional selection is more robust to mutations than the population after selection. The ratio of neighbor to parental activity decreases significantly from *R* = 0.84 to *R* = 0.82. This is visualized by the downward slope of the grey line plot in Figure 1A (see the right vertical axis). As we will see, this small decrease in robustness is consistent with the change we observed in the genotypic composition of the population. We note that the lines in Figure 1 are only used for visual aid, and we are using “slope” as a descriptor.

### The Decanalization is Caused by the Selective Sweep of a Low Robustness Variant

A candidate explanation for the observed decanalization in our population is that a short period of directional selection produced higher-fitness individuals, but these individuals suffered from decreased mutational robustness as compared to the canalized ancestors. To test this idea, we determined the mutational robustness of the wild-type ribozyme and the high fitness descendant genotype, Azo*. This variant arose in the same population that we analyzed above.

To determine the robustness of the Azo* genotype, we again used the mutagenic PCR protocol. We constructed two populations of mutational neighbors, one for the wild-type and one for Azo*. As expected, in both populations activity decreased relative to both wild-type (downward sloping blue line in Figure 1B) and Azo* (downward sloping red line). In both cases the decrease is significant (two-way ANOVA, P<10^−7^, n = 8). However, the decrease in activity is greater for Azo* than for the wild-type, that is, the lines have different slope (significant interaction in two-way ANOVA, P<10^−7^). Robustness, the activity ratio of mutants to the parental clone, equals *R* = 0.78 for the wild-type and *R* = 0.65 for Azo*, a decrease of ∼17%. Using only this data we can estimate that the robustness of the population would decrease by ∼4% during the period of directional selection when Azo* increased in frequency to represent 25% of the population (17% of 25% is about 4%). While the actual populations studied in Figure 1A are much more complex than a 3∶1 mixture of two genotypes, we conclude that the observed decrease in the robustness of the population as a whole can be mostly accounted for by the decreased robustness of Azo*.

### Co-evolution of Mutational and Environmental Robustness

Mutational robustness and environmental robustness may co-evolve [1], [4], [9], [30]–[33]. Supporting evidence in the context of RNA secondary structures exists from computational studies where stabilizing selection to maintain a single structure resulted in both increased mutational robustness and a type of thermodynamic stability (lower structural plasticity) [34]. Thermodynamic stability is a form of environmental robustness that buffers against temperature fluctuations. These observations suggested that the Azo* genotype might also be decanalized in terms of environmental robustness.

To test this hypothesis, we asked whether the Azo* genotype also has lower thermodynamic stability than the wild-type. The temperature at which structural elements of RNA unfold can be determined by plotting the derivative of UV absorbance (dA/dT) as a function of increasing temperature. This derivative data can then be fit to a thermodynamic model in order to identify the denaturation temperatures of base-paired segments, which appear as peaks in the derivative plot. Our thermal denaturation data confirm that Azo* has a destabilized secondary structure compared to the wild-type (Fig. 1C). The derivative melting profiles show two major denaturation transitions (peaks), consistent with earlier studies on the wild-type *Azoarcus* ribozyme [35]. The lower temperature transition (∼35°C) is not changed by the four Azo* mutations. However, these mutations do cause a significant decrease in the higher temperature transition (t-test P<10^−7^, wild-type n = 7; Azo* n = 8). The average denaturation temperature decreases from 51.6°C for the wild-type genotype down to 45.4°C for Azo*, a decrease of about 6°C.

### Conclusions

Directional selection caused decanalization in our evolving population of ribozymes. In general, decanalization that arises during directional selection would be caused by a selective sweep of new and superior genotypes, of which our Azo* genotype is an example. This genotype shows higher fitness, facilitating a selective sweep, but it also suffers from both decreased environmental and mutational robustness. These observations demonstrate that selective sweeps can cause decanalization and can do so even on short evolutionary time scales.

A computational study has suggested that RNA structures of ribozymes discovered through laboratory evolution have lower mutational robustness than naturally occurring structures [36]. Our experimental study is consistent with this suggestion. Canalized ribozymes might eventually rise to high frequency in our populations upon occurrence of further mutations, if our experiments were carried out for a sufficiently long time [37]. Also, the constant temperature of our laboratory experiments (37°C) relaxes selection for thermodynamic stability, as compared to the natural environments of *Azoarcus*, which include desert regions subject to extreme temperature fluctuations [38]. Our results support the idea that the short time scales and relaxed selection pressures of laboratory evolution experiments cause the selection of decanalized ribozymes. This suggests future avenues for preventing decanalization or promoting canalization in laboratory settings.

## Materials and Methods

### Previous Ribozyme Evolution

The evolution experiments that generated the populations of ribozymes from before and after directional selection were previously reported [28]. Briefly, directional selection was accomplished by amplifying variants that demonstrated activity in a reverse splicing assay containing 20 pmol RNA (10^13^ molecules), 30 mM EPPS (pH 7.5), 25 mM MgCl_2_, and 200 pmol oligonucleotide substrate with a phosphorothioate bond at the scissile phosphate (equal parts Rp/Sp). Reactions were allowed to proceed for 1 h at 37°C. About 20% of the reaction was subjected to reverse-transcription (AMV RT, Fermentas) and PCR (*Taq* polymerase, NEB) under the suppliers recommended conditions, and with a primer complementary to the 3′-end of the substrate (5′-TATTTATTTATTTATTTCC-3′), which becomes covalently attached to ribozymes that had successfully reverse spliced. Approximately 0.1 fmol (∼6×10^7^ molecules) of resulting PCR product was subjected to a second PCR to regenerate the active form of the ribozyme without the substrate derived sequence at the 3′-end, and to add the promoter sequence for T7 RNA polymerase to the 5′-end. To synthesize the next generation of ribozymes, this second PCR product was transcribed by T7 RNA polymerase (20 U, Fermentas) in 200 µL reactions containing 2 mM each nucleotide triphosphate (ATP, GTP, CTP, UTP), 50 mM Tris pH 7.5, 15 mM MgCl_2_, 5 mM DTT, 2 mM spermidine, and approximately 160 ng dsDNA template. Transcribed RNA was DNase treated (10 U RNase-free DNase I, Promega), extracted with phenol:chloroform (5∶1, pH 4.5, Ambion), ethanol precipitated, and PAGE purified to ensure length homogeneity. Excised RNA was eluted from the gel by diffusion into 0.3 M sodium acetate, filtered through a 0.2 micron centrifugal filter (Pall Life Sciences) then precipitated by the addition of ethanol. Precipitated RNA was rehydrated in RNase free water (Ambion) and a sample (2 µL) was quantified by UV absorbance (NanoDrop Technologies), and diluted in order to keep a constant population size (10^13^ molecules) at the beginning of each round of selection. We estimated the mutation rate of the entire process to be about 0.001 mutations per nucleotide per round of selection, and the length of the variable portion (inside the primer binding sites) of our ribozyme is 159 nucleotides [28].

The population resulting after eight rounds of this procedure is referred to here as “the population after directional selection”. The starting population, referred to here as “the population before directional selection”, resulted from ten rounds of the same procedure, except with a native RNA oligonucleotide substrate instead of a phosphorothioate bond containing substrate. During these initial rounds of selection with the native substrate, the second PCR was carried out under mutagenic conditions (see below), resulting in an elevated mutation supply rate of ∼0.007 per nucleotide per round [27], [28].

### Mutagenesis

T7 promoter containing dsDNA templates encoding the wild-type *Azoarcus* group I ribozyme and the Azo* variant were produced through a PCR-like assembly of synthetic deoxyoligonucleotides [39]. cDNA templates of populations of ribozymes were generated by reverse transcription during the evolution experiments described above. Non-mutated populations and clones were produced through high-fidelity PCR of these templates using VENT polymerase (NEB). Mutated populations (“neighbors”) were produced using a mutagenic PCR protocol [27]. The protocol was chosen because it was developed and optimized to allow gene randomization with very little nucleotide sequence bias. Based on the analysis of 16,591 nucleotides from two “biological” replicates, Cadwell and Joyce report the rates of each individual type of mutation, N to X and X to N (N = A, C, G, or T; X is not N). None of the rates were significantly different, except a slight preference for T to X changes, which are still less than twice as likely as any other N to X change. Further, it should be noted that this very small mutational bias occurs on either strand of the dsDNA templates during PCR, and the resulting RNA sequence is not expected to maintain this bias. Our populations are comprised of ∼197 nt long sequences with an average of ∼5 differences from wild-type (2.5%) for the population before directional selection, and ∼9 differences from wild-type for the population after directional selection [28]. Based on this, and the lack of mutational bias, it was concluded that different sequences in our populations were not expected to show significantly different mutation rates. PCR products were transcribed using T7 RNA polymerase and PAGE purified, as described above.

### Activity Assays

Activities of RNA samples were measured in 10 µL reactions containing 1 µM ribozyme, 5 µM RNA oligonucleotide substrate (Microsynth), 25 mM MgCl_2_, and 30 mM EPPS (pH 7.5). Reactions proceeded for 1 hour, and were stopped by the addition of 10 µL of formamide loading dye containing 25 mM EDTA. Reacted and unreacted ribozymes were separated on denaturing polyacrylamide gels and stained with GelRed (Biotum) for fluorescent quantification. Reacted ribozymes were distinguishable by an upward gel shift caused by the covalent ligation of a portion of the substrate to the 3′-end of the ribozyme. Activities were measured as the ratio of the fluorescence of bands representing unreacted and reacted ribozyme.

### UV Melting Curves

Thermal denaturation was carried out in 250 µL volumes containing 10 mM sodium cacodylate buffer (pH 7.5) and 0.1 µM RNA. The absorbance as a function of temperature was monitored during cooling from 95°C to 5°C at a rate of 0.5°C/min using a Cary 100 UV/Vis spectrophotometer. The 260 nm data was analyzed using the program Global Melt Fit (http://www.jhu.edu/~chem/draper).
